# Total hip arthroplasty with modular stem for Crowe I and II developmental dysplasia of the hip

**DOI:** 10.1186/s13018-019-1408-2

**Published:** 2019-11-13

**Authors:** Xiangpeng Kong, Yunming Sun, Minzhi Yang, Yonggang Zhou, Jiying Chen, Wei Chai, Yan Wang

**Affiliations:** 1 Department of Orthopaedics, Chinese PLA General Hospital, No.28 Fuxing Road, Haidian, Beijing China; 2 Department of Orthopaedics, Shengli Hospital of Shandong Dongying, No.31 Jinan Road, Dongying, Shandong China; 3 Nankai University, No.94 Weijin Road, Nankai, Tianjin China

**Keywords:** Hip dislocation, congenital, Arthroplasty, replacement, hip, Modular prosthesis, Leg length discrepancy, Offset, Forgotten joint score

## Abstract

**Background:**

The variation of femoral anteversion is not completely consistent with the grade of developmental dysplasia of the hip (DDH), which poses challenges to hip replacement with the non-modular tapered stem. Currently, whether the modular stem should be used in Crowe I and II DDH is still controversial. The aim of this study is to compare the clinical efficacy of the modular stem and the non-modular tapered stem in Crowe I and II DDH patients.

**Methods:**

We retrospective analyzed the clinical data of 196 patients with unilateral Crowe I and II DDH from January 2015 to January 2017. One hundred patients were operated by an experienced surgeon with the modular stems; the remaining 96 patient was operated by another equivalent surgeon with the non-modular tapered stems. The preoperative basic information, operating time, intraoperative and postoperative complications, postoperative leg length discrepancy (LLD) and offset, Harris hip score (HHS), and forgotten joint score (FJS) in postoperative 2 years were collected and analyzed.

**Results:**

Postoperative LLD (*P* = 0.010) and FJS (*P* = 0.001) had significant difference between two groups. Concurrent acceptable LLD and offset were achieved in 87% of patients with the modular stem and in 68% of patients with the non-modular stem (*P* = 0.001). There was no significant difference in the operating time (*P* = 0.086), intraoperative complication (*P* = 0.096), postoperative dislocation rate (*P* = 0.056), postoperative offset difference (*P* = 0.108), and Harris score (*P* = 0.877) between two groups.

**Conclusions:**

Compared with the non-modular tapered stem, the modular stem was more likely to provide accurate reconstruction and forgotten artificial hip for Crowe I and II DDH patients. We recommend the modular stem as routine choice for these patients.

## Background

Total hip arthroplasty (THA) has been one of the most mature orthopedic surgery in recent years [1, 2].

Its clinical outcome is encouraging, but not perfect. Leg length discrepancy (LLD), altered hip biomechanics, dysfunctional gait, lower back pain, instability, and dislocation followed by THA are recognized as imperfections or complications [3, 4]. In America, postoperative LLD has been the leading factor in patient dissatisfaction and litigation [5]. Besides the surgical technique, the design of prosthesis also plays an important role in the restoration of the normal biomechanics [6]. The femoral stem with two modular junctions was proved to have more frequent ability to restore femoral offset and leg length than the single modular junction [7].

Developmental dysplasia of the hip (DDH) is the most leading reason of secondary osteoarthritis of the hip [8, 9]. Considerable studies indicate the morphology of the dysplastic femur has features of abnormal anteversion and narrow medullary [10–12]. Additionally, morphological parameters were found not to be statistically correlated with the severity of hip dislocation [13]. Because of time-consuming, high-cost, and substantial radiation, computerized tomography (CT) was not always available though it allows for accurate assessments of anteversion [14]. These hidden abnormalities in DDH pose big challenges to THA.

In the past, a large number of studies focused on DDH, but most of them were restricted to Crowe III and IV DDH. Given the unpredictable variation in Crowe I and II DDH, more attention should be paid to these patients. Currently, whether the modular stem should be used in Crowe I and II DDH was still controversial [15–17]. We have reported the short-term clinical outcomes of the modular stem previously [18]. In this study, we try to find the answers of the following questions: (1) whether the use of the modular stem could restore limb length and offset more accurately than the non-modular tapered stem and (2) whether the stem’s modularity could provide the patients with better function and less complication.

## Patients and methods

We respectively analyzed the patients with unilateral Crowe I and II DDH in our joint registry system from January 2015 to January 2017. The S-ROM (modular stem, Depuy, Warsaw, USA) and LCU (non-modular tapered stem, Link, Hamburg, Germany) were used for primary THA in our institute. As one modular stem, S-ROM includes sleeve (triangle and cone) and straight stem. The sleeve can be rotated by 360° of version. It has three kinds of neck lengths, and every neck length has two kinds of offset. LCU is one kind of tapered stem with unified neck length and offset.

Inclusion criteria include the following: (1) the surgeries were performed by the certain two senior surgeons through posterolateral approach, (2) the contralateral hip was normal, (3) the follow-up time was > 2 years. The patients who had previous hip surgery and incomplete medical data were excluded. The study was approved by the institutional review board. Finally, a total of 196 patients was enrolled in this study, which included 100 patients in the group of S-ROM and 96 patients in the group of LCU. The surgeons aimed to place the acetabular cup at 20° anteversion and 40° abduction. When the leg length (comparing the distal polar of patella) and joint stability (flexion 90° and internal rotation 45°, extension 0°, and maximum external rotation) were satisfying, the surgeon implanted the true femoral stem and femoral head. The external rotating muscles and capsule were sutured to the greater trochanter of the femur.

All patients received antibiotics within 24 h and aspirin in 35 days postoperatively. The patients had regular follow-ups at 3 months, 1 year, and 2 years after operation.

Preoperative and postoperative LLD and offset, operating time, intraoperative complications (femoral fracture, temporary replacement of the femoral stem), postoperative complications (prosthetic loosening, dislocation), Harris hip score (HHS), and forgotten joint score (FJS) were recorded.

LLD was measured by drawing the bilateral teardrops’ connecting line and measuring a perpendicular line to the lesser trochanters. The absolute value of the difference between the distances was the LLD. The metal marker and acetabular cup were used to adjust the imaging error. If the teardrops were poorly visible, the ischial tuberosity was used. If the lesser trochanters were poorly visible, the greater trochanters were used. The LLD < 10 mm was recorded as acceptable [19].

Offset was measured as the distance from the inferior border of the teardrops to the long axis of the femoral shaft. The absolute value of the difference between bilateral offsets was the offset’s discrepancy (offset-D). The offset-D < 5 mm was recorded as acceptable [20].

Data were analyzed using the SPSS 15.0 and *P* < 0.05 denoted a significant difference. Measurements such as Harris score and FJS were expressed as *x ± s*. Measurement data was analyzed by *t* test or analysis of variance. Count data were analyzed by *χ*^*2*^ test or Fisher’s exact test.

## Results

There were no significant differences in preoperative demographic data between the two groups (*P* > 0.05, Table 1).

**Table 1:** Comparison of preoperative demographic data between the two groups

Group	Cases	Crowe I/II	Gender (male/female)	Age (*x* ± *s*, years)	Height (*x* ± *s*, cm)	Weight (*x* ± *s*, kg)	BMI (*x* ± *s*, kg/m^2^)
S-ROM	100	37/63	6:94	42.42 ± 11.18	157.28 ± 8.00	58.40 ± 10.02	23.69 ± 5.45
LCU	96	30/66	5:91	39.74 ± 11.61	159.37 ± 10.24	59.74 ± 12.51	23.63 ± 5.70
*P*	–	0.396	0.810	0.273	0.130	0.405	0.963

### LLD and offset

Regarding preoperative measurement and clinical function, there were no significant differences in LLD and offset-D between the two groups. Both groups had significant improvement in LLD and offset (Table 2). Postoperative LLD in the group of S-ROM was significantly lower than that in the group of LCU (3.80 ± 3.13, 5.45 ± 3.88, *P* = 0.042). The rate of acceptable LLD in the group of S-ROM was also significantly higher than that in the group of LCU (92% vs 79%, *P* = 0.010). The rate of concurrent acceptable LLD and offset-D in the group of S-ROM was significantly higher than that in the group of LCU (87% vs 65%, *P* = 0.001). The offset and rate of acceptable offset in the group of S-ROM were more normal than that in the group of LCU, but the differences were not significant (Table 3).

**Table 2:** Comparison of preoperative and postoperative clinical outcome between the two groups

Group	HHS (*x* ± *s*)			LLD			Offset-D			FJS
	Pre-operation	Post-operation	*P*	Pre-operation	Post-operation	*P*	Pre-operation	Post-operation	*P*	
S-ROM	48.58 ± 16.29	87.46 ± 8.53	0.000	12.18 ± 5.43	3.80 ± 3.13	0.000	6.13 ± 3.44	2.80 ± 2.13	0.000	90.08 ± 7.29
LCU	51.25 ± 14.87	87.40 ± 10.80	0.000	11.01 ± 4.90	5.45 ± 3.88	0.000	5.21 ± 2.91	3.95 ± 1.88	0.001	86.42 ± 8.42
*P*	0.232	0.877	–	0.767	0.042	–	0.560	0.072	–	0.001

**Table 3:** Comparison of intraoperative and postoperative data between the two groups

Group	Case	Operating time	Intraoperative complications	Postoperative outcomes			
				Dislocation	Acceptable LLD (< 10 mm)	Acceptable offset-D (< 5 mm)	Concurrent acceptable LLD and offset-D
S-ROM	100	67.38 ± 12.51	2 (2%)	0 (0%)	92 (92%)	91 (91%)	87 (87%)
LCU	96	62.66 ± 13.34	7 (7%)	4 (4%)	76 (79%)	80 (83%)	65 (68%)
*P*	–	0.086	0.096	0.056	0.010	0.108	0.001

### Postoperative function

In the postoperative 2 years, although there was no significant difference in HHS between two groups, FJS in the group of S-ROM was significantly higher than that in the group of LCU (90.08 ± 7.29, 86.42 ± 8.42, *P* = 0.001). These results indicated that S-ROM could provide higher FJS for the patients through restoring the normal leg length and offset.

### Intraoperative complications

**Fig. 1:**
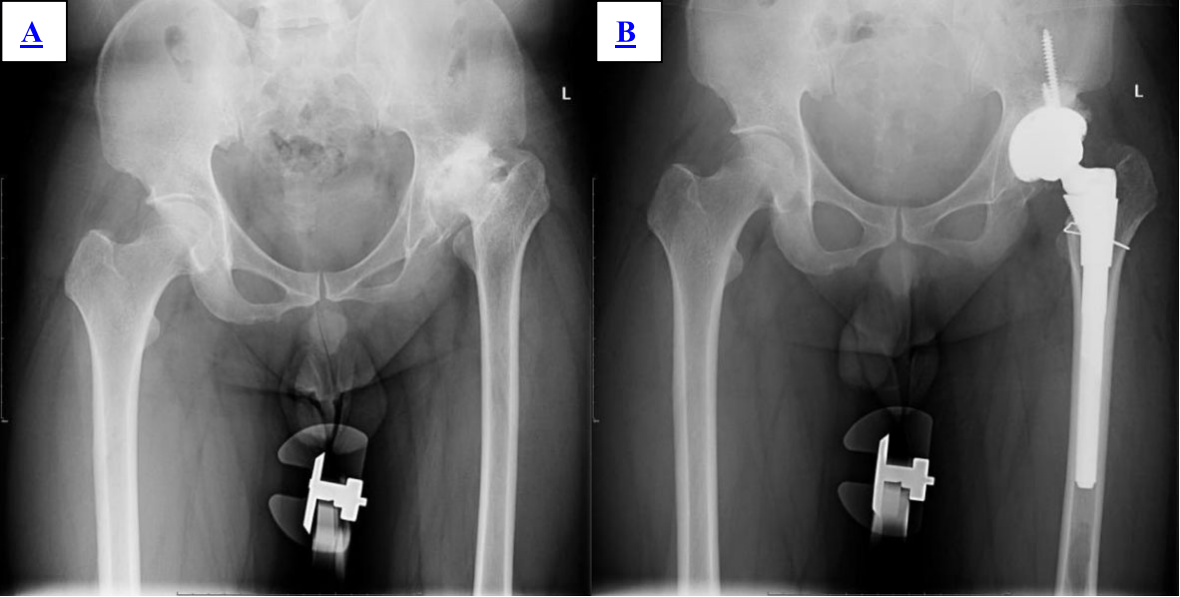
Male, 52 years old. Due to poor stability of LCU trial, the surgeon changed to S-ROM intraoperatively. **a** Preoperative X-ray showed left Crowe II DDH. **b** Postoperative X-ray

Although the operating time of S-ROM was longer than that of LCU, The difference was not significant (67.38 ± 12.51 min, 62.66 ± 13.34 min, *P* = 0.086). In the group of S-ROM, two patients had periprosthetic fractures of proximal femur during the operation. In the group of LCU, three patients had a periprosthetic fracture of the proximal femur and one patient had a periprosthetic fracture of the distal stem during the operation. All of them were required to delay the loading time and got healed eventually. Three patients in the group of LCU were transferred to S-ROM intraoperatively because of excessive anteversion (Fig. 1) (Table 3).

### Postoperative complications

**Fig. 2:**
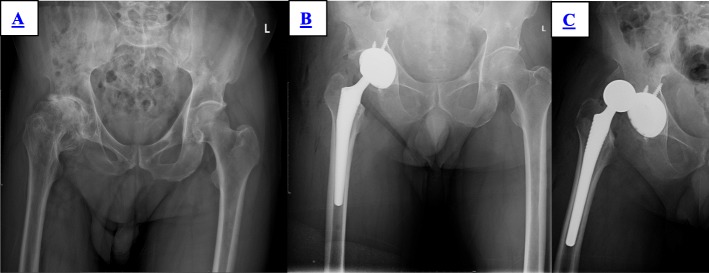
Male, 38 years old. **a** Preoperative X-ray showed right Crowe I DDH. **b** Postoperative X-ray. **c** Anterior dislocation in the 1st day postoperatively

**Fig. 3:**
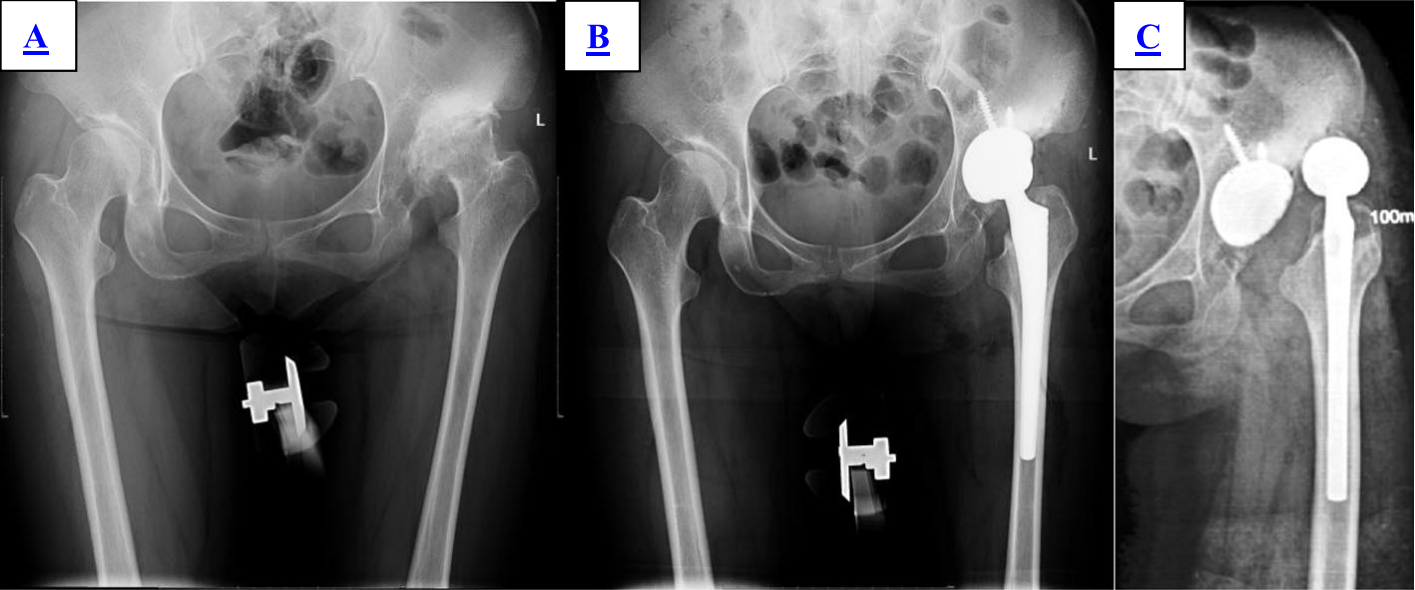
Female, 38 years old. **a** Preoperative X-ray showed left Crowe II DDH. **b** Postoperative X-ray. **c** Anterior dislocation in the fourth day postoperatively

In the group of S-ROM, there was no dislocation during the 2 years of follow-up. In the group of LCU, four patients had dislocation in the first, fourth, fifth, and 11th day after operation, including three cases of anterior dislocation and one case of posterior location (Fig. 2). All four patients had manual reduction; the brace or anti-rotation shoes were worn for 3 months depending on the patient’s condition (Fig. 3). There were no re-dislocation, periprosthetic infections, or revisions among all the patients until the last follow-up (Table 3).

## Discussion

The modular stems have proved to have excellent clinical outcome and satisfaction rate in severe DDH [14, 21]. The most important finding of this study was that the modular stem could provide more accurate hip reconstruction and better FJS for the patients with Crowe I and II DDH in postoperative 2 years.

Accurate hip geometry reconstruction has an important influence on clinical outcomes [19]. One previous study showed that when the LLD is less than 5 mm, the human generally does not feel the discrepancy. When the LLD is 5–10 mm, the human can sense the difference, but the sense would be gradually corrected through the compensation of the spine and pelvis within a certain period of time. When the LLD is larger than 10 mm, it is beyond the human’s compensatory ability and the sense will persist for a long time [22, 23].

In this study, acceptable LLD (< 10 mm) was achieved in 92% of patients with the modular stem and in 79% with non-modular tapered stem. The results have clearly reflected the advantage of the modular stem in controlling LLD.

Offset, not the leg length was usually compromised to achieve joint stability in clinical practice. Little et al. reported that the femoral offset was associated with the acetabular wear and the significant offset-D (> 5 mm) wound lead to accelerated wear [20]. Furthermore, offset and leg length had an additive influence on postoperative improvement in clinical outcome after total hip arthroplasty [19]. The hip reconstruction and biomechanics would be influenced, regardless of acceptable offset or acceptable LLD. So we should pay more attention to the balance of leg length and offset in primary THA. In this study, concurrent acceptable LLD and offset were achieved in 87% of patients with the modular stem and in 68% of patients with the non-modular tapered stem. The modularity of SROM provides the surgeon with more choices to reconstruct the dysplastic hip perfectly.

We further analyzed the reasons behind the above results. Firstly, because of the congenital abnormality of the acetabulum and femur, the surgeon had to change the anteversion of the acetabular cup to achieve enough coverage. The safe zone of the combined anteversion was difficult to reach with the non-modular tapered stem, even for those patients who had nearly normal femoral morphology. Secondly, when the non-modular tapered stem was used to correct the intrinsic femoral anteversion, the risk of fracture would increase. The limited ability of adjusting anteversion would also add the incidence of dislocation or unwanted leg lengthening. These potential disadvantages limit the application of the non-modular stem theoretically in dysplastic hips.

The modular stem might be a good choice. One previous study evaluated the three-dimensional anatomy of the femur with congenital dysplasia of the hip in comparison with healthy controls. This study showed that there was a significant difference in the geometry between the normal and dysplastic hips, even in mild cases. The authors recommended the use of modular or specially designed components to accommodate the shape of the dysplastic canal [24]. Another study enrolled 220 cases of hip replacement in DDH patients with S-ROM. These hips included 154 hips in Crowe I, 41 in Crowe II, 13 in Crowe III, and 12 in Crowe IV. The version of the stem was decreased against the sleeve by up to 60° in 56% of the hips, while the version was increased against the sleeve by up to 45° in 18% of the hips [21]. So the mechanism of free adjusting femoral anteversion was necessary in dysplastic hips. In addition, S-ROM has several options of neck length and offset, which enable the surgeons to achieve the triple win for stability, leg length, and offset.

Some surgeons questioned whether the subtle improvement of LLD and offset would perfect the clinical outcomes [17, 25]. Our study compared the surgical complications, hip function, and forgotten joint score to answer the question.

In the group of LCU of this study, four patients had a periprosthetic femoral fracture and another four patients had postoperative dislocation. However, there were only two patients who had a periprosthetic femoral fracture and no dislocation. Due to poor stability with LCU, three patients changed the surgical plan and transferred to S-ROM intraoperatively. Although the differences were not significant, the role of S-ROM in reducing dislocation and fracture might be proved with a larger sample size.

It is worth noting that higher FJS was achieved in patients with the modular stem than the non-modular tapered stem. The more natural hip might be related to better biomechanics, which probably involved with more accurate leg length and offset [21].

There were several limitations to this study. Firstly, the study was retrospective and the follow-up time was relatively short. The long-term hip function and prosthetic survivorship need to be further studied. Secondly, this study enrolled two senior surgeons and two kinds of femoral prostheses in one institute. While the two surgeons may have different surgical habits, similar surgical philosophies and strategies increased the comparability of the two groups. The S-ROM and LCU cannot fully represent the modular and non-modular prostheses. Thirdly, because there was no preoperative CT, the femoral anteversion of the patients in two groups was impossible to be measured accurately. Fourthly, this study did not take the economic factors and cost performance into consideration. The complex surgical procedures of the modular stem did increase the operating time and anesthetic cost. And its own higher price also would increase the financial burden of patients. Whether the extra expense could be offset by reduced complications need further study.

## Conclusions

Compared with the non-modular tapered stem, the modular stem was more likely to provide accurate reconstruction and forgotten artificial hip for Crowe I and II DDH patients. We recommend the modular stem as a routine choice for these patients.

## Data Availability

All data generated or analyzed during this study are included in this published article.

## Abbreviations

BMI: Body mass index
CT: Computerized tomography
DDH: Developmental dysplasia of hip
FJS: Forgotten joint score
HHS: Harris hip score
LLD: Leg length discrepancy
offset-D: Offset’ s discrepancy
THA: Total hip arthroplasty
