# Correction to: Total hip arthroplasty with modular stem for Crowe I and II developmental dysplasia of the hip

**DOI:** 10.1186/s13018-019-1501-6

**Published:** 2019-12-17

**Authors:** Xiangpeng Kong, Yunming Sun, Minzhi Yang, Yonggang Zhou, Jiying Chen, Wei Chai, Yan Wang

**Affiliations:** 1 Department of Orthopaedics, Chinese PLA General Hospital, No.28 Fuxing Road, Haidian, Beijing China; 2 Department of Orthopaedics, Shengli Hospital of Shandong Dongying, No.31 Jinan Road, Dongying, Shandong China; 3 Nankai University, No.94 Weijin Road, Nankai, Tianjin China

**Correction to: J Orthop Surg Res**

**https://doi.org/10.1186/s13018-019-1408-2**

In the original publication of this article [1], an sentence needs to be added in the end of the caption of Figure 1 and Figure 3: cited in our previous study [18].

## References

[1] Kong (2019). Total hip arthroplasty with modular stem for Crowe I and II developmental dysplasia of the hip. J Orthop Surg Res.

